# Elf3 Contributes to Cartilage Degradation *in vivo* in a Surgical Model of Post-Traumatic Osteoarthritis

**DOI:** 10.1038/s41598-018-24695-3

**Published:** 2018-04-24

**Authors:** Elisabeth B. Wondimu, Kirsty L. Culley, Justin Quinn, Jun Chang, Cecilia L. Dragomir, Darren A. Plumb, Mary B. Goldring, Miguel Otero

**Affiliations:** 1 HSS Research Institute, Hospital for Special Surgery, New York, NY 10021 USA; 2 Weill Cornell Graduate School of Medical Sciences, New York, NY 10021 USA; 3 Department of Cell and Developmental Biology, Weill Cornell Medical College, New York, NY 10021 USA

## Abstract

The E-74 like factor 3 (ELF3) is a transcription factor induced by inflammatory factors in various cell types, including chondrocytes. ELF3 levels are elevated in human cartilage from patients with osteoarthritis (OA), and ELF3 contributes to the IL-1β-induced expression of genes encoding *Mmp13*, *Nos2*, and *Ptgs2/Cox2* in chondrocytes *in vitro*. Here, we investigated the contribution of ELF3 to cartilage degradation *in vivo*, using a mouse model of OA. To this end, we generated mouse strains with cartilage-specific *Elf3* knockout (*Col2*Cre:*Elf3*^f/f^) and *Comp*-driven Tet-off-inducible *Elf3* overexpression (TRE-*Elf3*:*Comp*-tTA). To evaluate the contribution of ELF3 to OA, we induced OA in 12-week-old *Col2*Cre:*Elf3*^f/f^ and 6-month-old TRE-*Elf3*:*Comp*-tTA male mice using the destabilization of the medial meniscus (DMM) model. The chondrocyte-specific deletion of *Elf3* led to decreased levels of IL-1β- and DMM-induced *Mmp13* and *Nos2* mRNA *in vitro* and *in vivo*, respectively. Histological grading showed attenuation of cartilage loss in *Elf3* knockout mice compared to wild type (WT) littermates at 8 and 12 weeks following DMM surgery that correlated with reduced collagenase activity. Accordingly, *Elf3* overexpression led to increased cartilage degradation post-surgery compared to WT counterparts. Our results provide evidence that ELF3 is a central contributing factor for cartilage degradation in post-traumatic OA *in vivo*.

## Introduction

Osteoarthritis (OA) is a whole joint disorder characterized by pathologic changes of multiple components of the joint^1,2^. Although OA has long been regarded as “wear and tear” arthritis, the involvement of inflammation in disease progression has been well recognized in recent years^2,3^. Inflammatory and mechanical stress in OA leads to abnormal activation of common signaling pathways in the cells resident in joint tissues^4,5^. Chondrocytes, constituting the unique cell type of articular cartilage, become activated in OA in response to the abnormal environment, leading to a plethora of transcriptional and non-transcriptional events that result in aberrant expression of genes contributing to the degradation of the extracellular matrix^6^.

The ETS transcription factors, defined by a highly conserved DNA binding domain^7^, are implicated in a diverse range of biological functions^8,9^. The E-74 like factor 3 (ELF3) is a member of the ELF subfamily with roles in epithelial cell differentiation and function^10,11^. ELF3 is abundantly expressed in epithelial tissues^10^. In non-epithelial cells, in which it is not expressed normally^12,13^, the induction of ELF3 by inflammatory stimuli drives the expression of genes such as cyclooxygenase 2 (*COX2*), nitric oxide synthase 2 (*NOS2*), angiopoietin-1, and matrix metalloproteinase 13 (*MMP13*)^12,14–17^.

ELF3 is expressed in the cartilage and synovium of patients with rheumatoid arthritis and OA^12,17,18^. We showed that ELF3 represses the expression of the type II collagen gene (*COL2A1*) by directly binding to the *COL2A1* promoter^18^ and disrupting the activator functions of Sox9 and CBP/p300^19^. In addition, we showed that ELF3 binds to and transactivates the *MMP13* promoter in chondrocytes, thereby mediating IL-1β- and TNFα-driven *MMP13* gene expression^17^. Altogether, these results suggest that ELF3 plays a critical role in cartilage remodeling in OA disease.

In this study, we aimed to investigate the contribution of ELF3 to cartilage degradation *in vivo* in a model of post-traumatic OA. Using mice with *Col2a1*-driven *Elf3* knockout (Elf3-cKO) or *Comp*-driven Tet-Off inducible overexpression of *Elf3* (Elf3-Tg) subjected to destabilization of the medial meniscus (DMM), we show that *Elf3* accounts, in large part, for the expression and activity of *Mmp13* in articular chondrocytes *in vivo*.

## Results

### Generation and characterization of cartilage-specific *Elf3* KO mice

**Figure 1:**
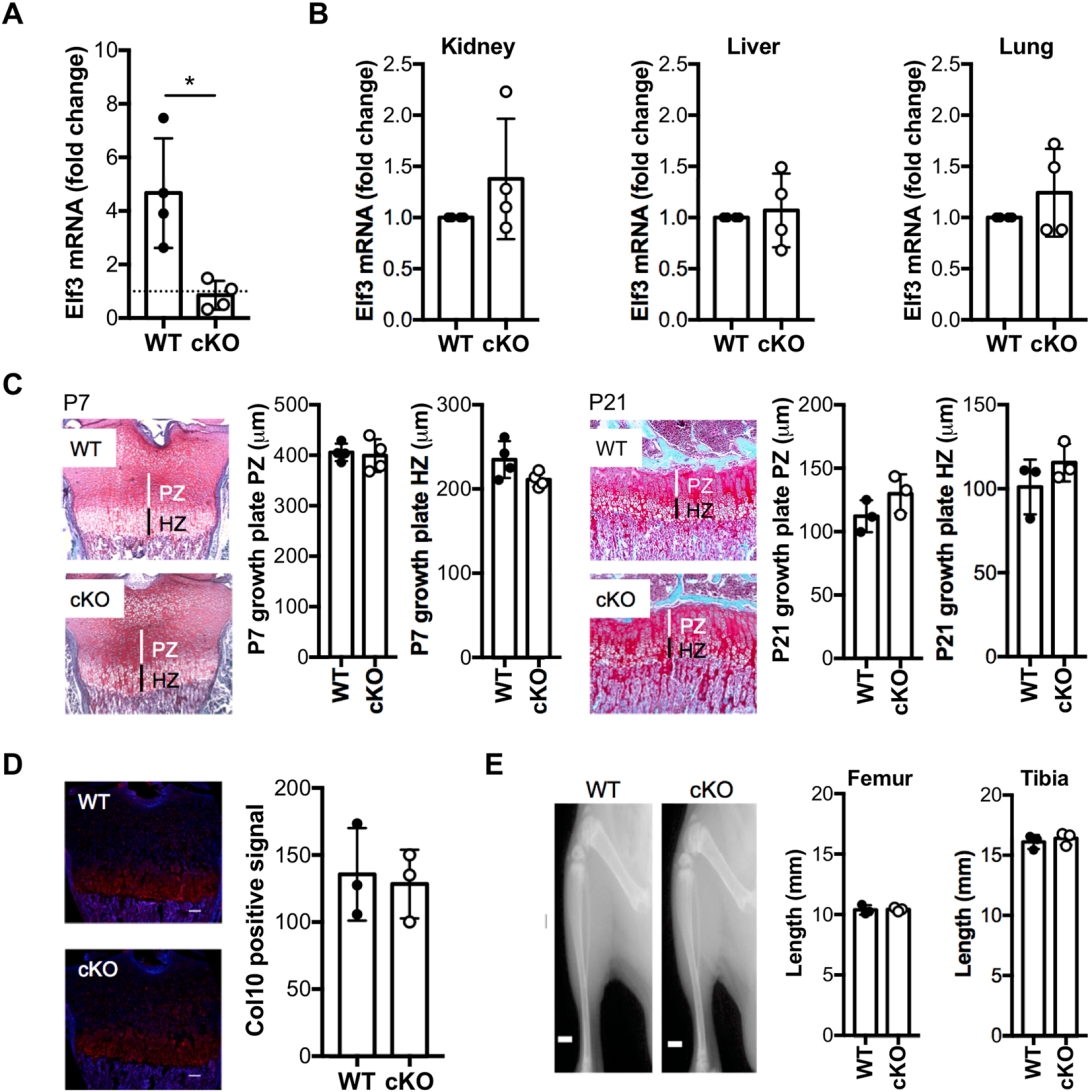
Characterization of mice with cartilage-specific Elf3 deficiency. (**A**) IL-1β treatment significantly induced *Elf3* mRNA in WT but not in cKO cartilage explants (n = 4/ea). Data are represented as the fold-change in mRNA levels relative to untreated controls (dotted line). (**B**) RTqPCR analyses of total RNA extracted from kidney, liver, and lung (n = 4/ea) showed no difference in *Elf3* mRNA levels between cKO and WT samples. (**C**) Representative histological sections of growth plates from tibiae of cKO and WT mice at P7 and P21. Vertical lines indicate proliferative (PZ, white) and hypertrophic (HZ, black) zones. Quantification of the thickness of hypertrophic and proliferative zones is shown for P7 (n = 4/genotype) and P21 (n = 3/genotype). (**D**) Representative images of type X collagen (Col10) immunostaining in hypertrophic zones of cKO and WT mice at P7. The quantification of the Col10-positive area (n = 3/ea) is shown as mean signal intensity (red = Col10-positive, blue = DAPI). Scale bar = 100 μm. (**E**) Representative digital radiographs of tibiae and femora from WT and cKO mice at P21 (n = 3/ea). Quantification is shown on the right. Scale bar = 1.87 mm. Data are shown as mean ± S.D. *p < 0.05 by *t*-test.

To elucidate the role of *Elf3* deficiency in articular cartilage remodeling *in vivo*, we first generated mice with cartilage-specific *Elf3* KO. The *Col2a1*-Cre:*Elf3*^*f*/f^ (Elf3-cKO) mice were born at the expected Mendelian ratio, with no gross abnormality, and with comparable size and weight to the *Elf3*^*f/f*^ Cre-negative littermates (WT) and sex- and age-matched wild-type C57/BL6 mice (not shown). To assess knockout efficiency, total RNA was isolated from cartilage explants from femoral heads of 5- to 6-day-old cKO and WT littermates. Since Elf3 mRNA is barely detectable in articular chondrocytes and other non-epithelial cells^12,15,17^, we incubated the cartilage explants with 10 ng/ml IL-1β for 6 days. RTqPCR analyses confirmed increased *Elf3* mRNA in IL-1β-treated WT cartilage explants, whereas IL-1β treatment did not induce *Elf3* mRNA in cKO cartilage explants, verifying efficient *Elf3* ablation in articular cartilage (Fig. 1A). Next, to verify the cartilage specificity of *Elf3* deletion, we assessed *Elf3* mRNA levels in tissues known to express *Elf3* at baseline. As shown in Fig. 1B, E*lf3* mRNA levels were similar in kidney, liver, and lung tissues obtained from cKO compared to WT littermates. Our preliminary *in situ* hybridization analyses showed the absence of *Elf3* mRNA in the developing cartilage anlagen of C57/BL6 mice (not shown), suggesting that Elf3 does not contribute to growth plate development. To confirm this, we assessed whether the postnatal growth plate architecture was altered in the cKO animals. As shown in Fig. 1C, we observed no difference in the lengths of the post-natal proliferating and hypertrophic zones between WT and cKO mice at P7 or P21. Further, immunofluorescence analyses in P7 (Fig. 1D) and P21 (not shown) mice revealed comparable distribution of type X collagen protein in the hypertrophic zone of WT and cKO mice. Finally, Faxitron radiographic analyses revealed no difference in the lengths of long bones (tibiae and femora) of WT and cKO mice at P7 (not shown) and P21 (Fig. 1E). Together, these results indicate that *Col2a1*-driven *Elf3* knockout does not lead to skeletal abnormalities, and that the adult *Elf3* cKO mice are suitable for studying OA.

### Decreased expression of *Elf3* target genes in primary chondrocytes from *Elf3*-deficient mice

**Figure 2:**
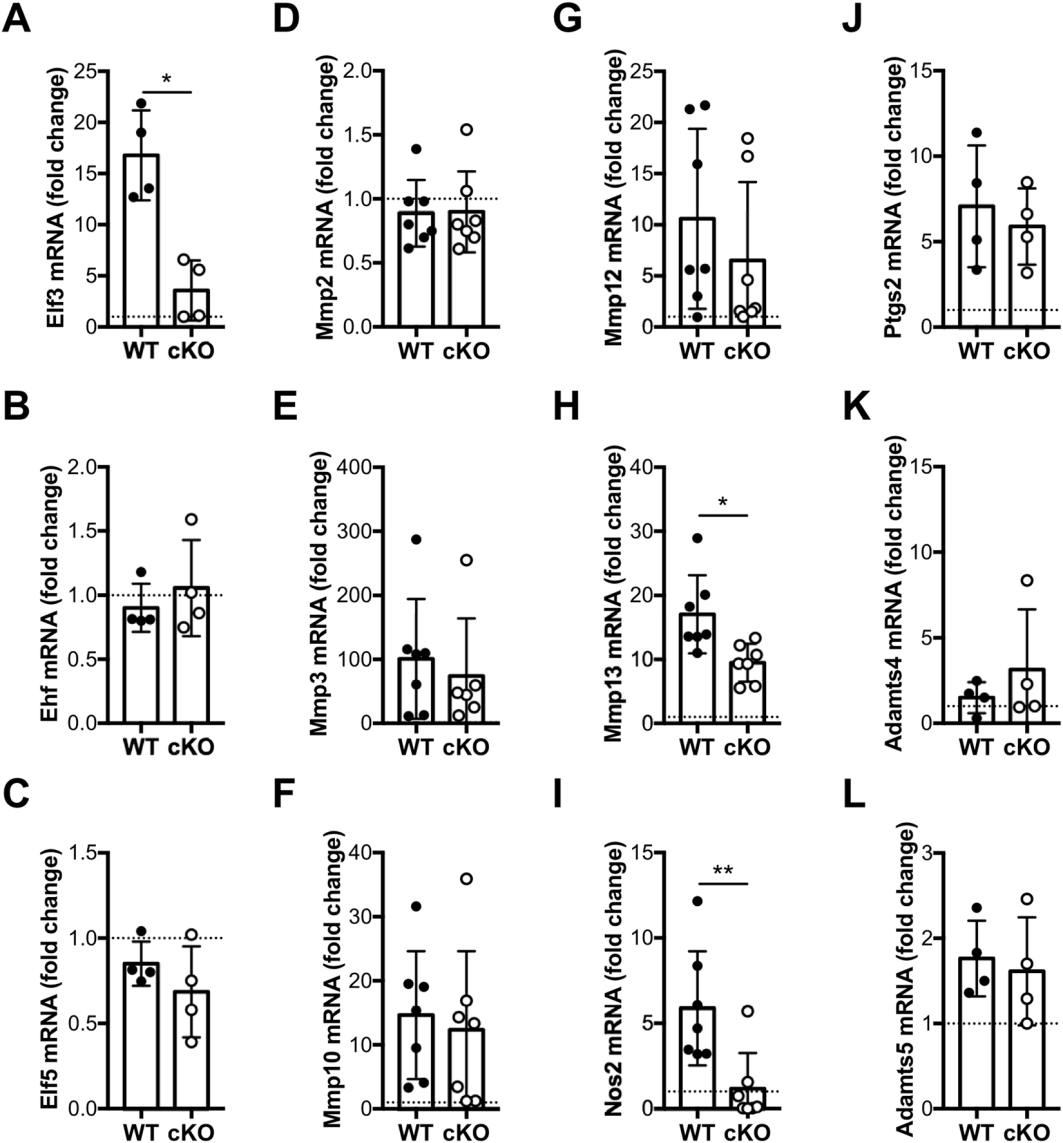
Decreased expression of IL-1β-induced Elf3 target genes in primary chondrocytes from Elf3-cKO mice. Primary chondrocytes isolated from 5- to 6-day-old WT and cKO littermates were left untreated (vehicle, PBS/BSA) or treated with IL-1β (1 ng/ml) for 6 h. The mRNA levels of Elf3 (**A**), Ehf (**B**), Elf5 (**C**), Mmp2 (**D**), Mmp3 (**E**), Mmp10 (**F**), Mmp12 (**G**), Mmp13 (**H**), Nos2 (**I**), Ptgs2 (**J**), Adamts4 (**K**), and Adamts5 (**L**) were analyzed by RTqPCR and are represented as fold-change vs. untreated controls (dotted line). Data are mean ± S.D. from at least 4 independent experiments. *p < 0.05, ** < 0.01 by *t*-test.

We next assessed the functional impact of cartilage-specific *Elf3* deletion by RTqPCR analyses of total RNA extracted from WT and cKO primary chondrocytes after treatment with 1 ng/ml of IL-1β for 6 hours *in vitro*. As shown in Fig. 2A, IL-1β-induced *Elf3* expression was ablated in cKO cells without impacting the levels of *Ehf* and *Elf5* mRNA, other members of the ELF subfamily (Fig. 2B,C). Importantly, while *Elf3* KO did not impact the basal or IL-1β-driven induction of *Mmp2, 3*, 10, or *12, Ptgs2*, or *Adamts4* or 5 mRNA, we observed a significant reduction in the IL-1β-driven *Nos2* and *Mmp13* gene expression (Fig. 2D–L). Together, our results confirmed our previous findings in chondrocytes from global *Elf3* KO mice^17^ and further suggested that ELF3 could contribute to cartilage remodeling in OA by driving collagenase gene expression.

### Cartilage-specific *Elf3* deficiency attenuates the development of osteoarthritis in mice following destabilization of the medial meniscus

**Figure 3:**
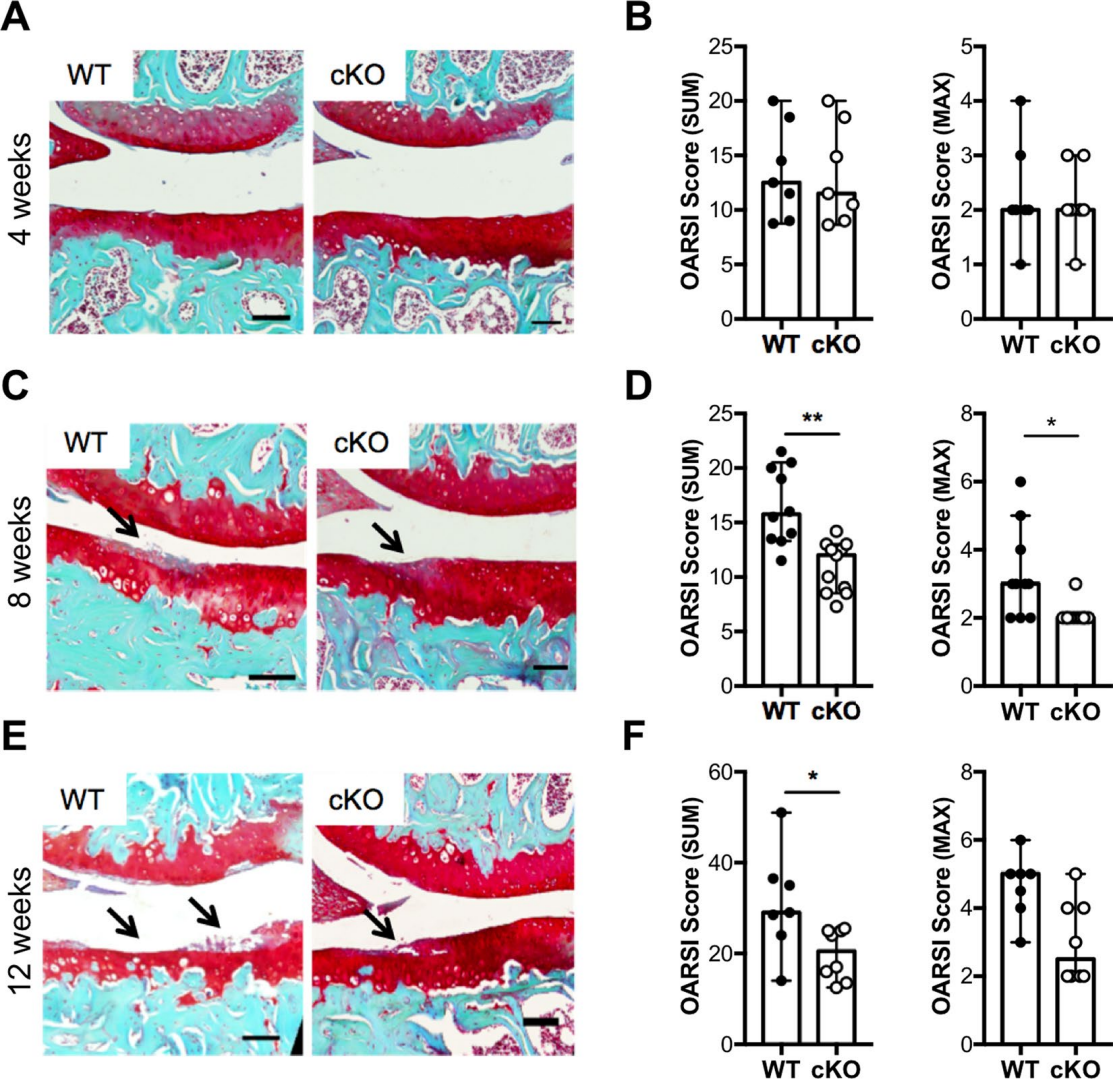
Cartilage-specific Elf3 deficiency attenuates the development of osteoarthritis in mice following destabilization of the medial meniscus (DMM). Representative Safranin O/Fast green-stained sections of the knee joints of DMM-operated 12-week-old male WT and cKO mice at (**A**) 4 weeks (WT = 7; cKO = 7), (**C**) 8 weeks (WT = 10; cKO = 11), and (**E**) 12 weeks (WT = 7; cKO = 8). Images show the medial femorotibial compartment of DMM-operated knees using 10X magnification. The arrows indicate sites of cartilage lesions. Quantification of cartilage damage in WT and cKO mice at 4 (**B**), 8 (**D**), and 12 (**F**) weeks following DMM is shown as OARSI SUM and MAX scores and represented as median and C.I. *p < 0.05 and **p < 0.01 by Mann-Whitney test.

Next, to determine whether ELF3 contributes to OA progression *in vivo*, we compared cartilage degradation in WT and cKO mice after surgical induction of OA. Articular cartilage and joint morphology were not different in cKO mice compared to WT littermates at baseline, in unoperated knees (Supplementary Figure 2). At 4 weeks post-DMM, small fibrillations were observed in both WT and cKO articular cartilage, with no significant difference in the OA scores (Fig. 3A,B). However, at 8 and 12 weeks post-surgery, cartilage damage was significantly reduced in the cKO mice (Fig. 3C–F). We also analyzed the size and maturity of osteophytes, as described^20^. The unoperated knees did not form osteophytes, as expected, and we did not observe any difference in osteophyte formation, size, or maturity between genotypes at 8 weeks following surgery (Supplementary Figure 3). These results indicate that cartilage-specific *Elf3* deletion impacts cartilage degradation without affecting osteophyte formation in a surgical model of post-traumatic OA.

### *Elf3* deficiency reduces collagenase activity in mice following destabilization of the medial meniscus

**Figure 4:**
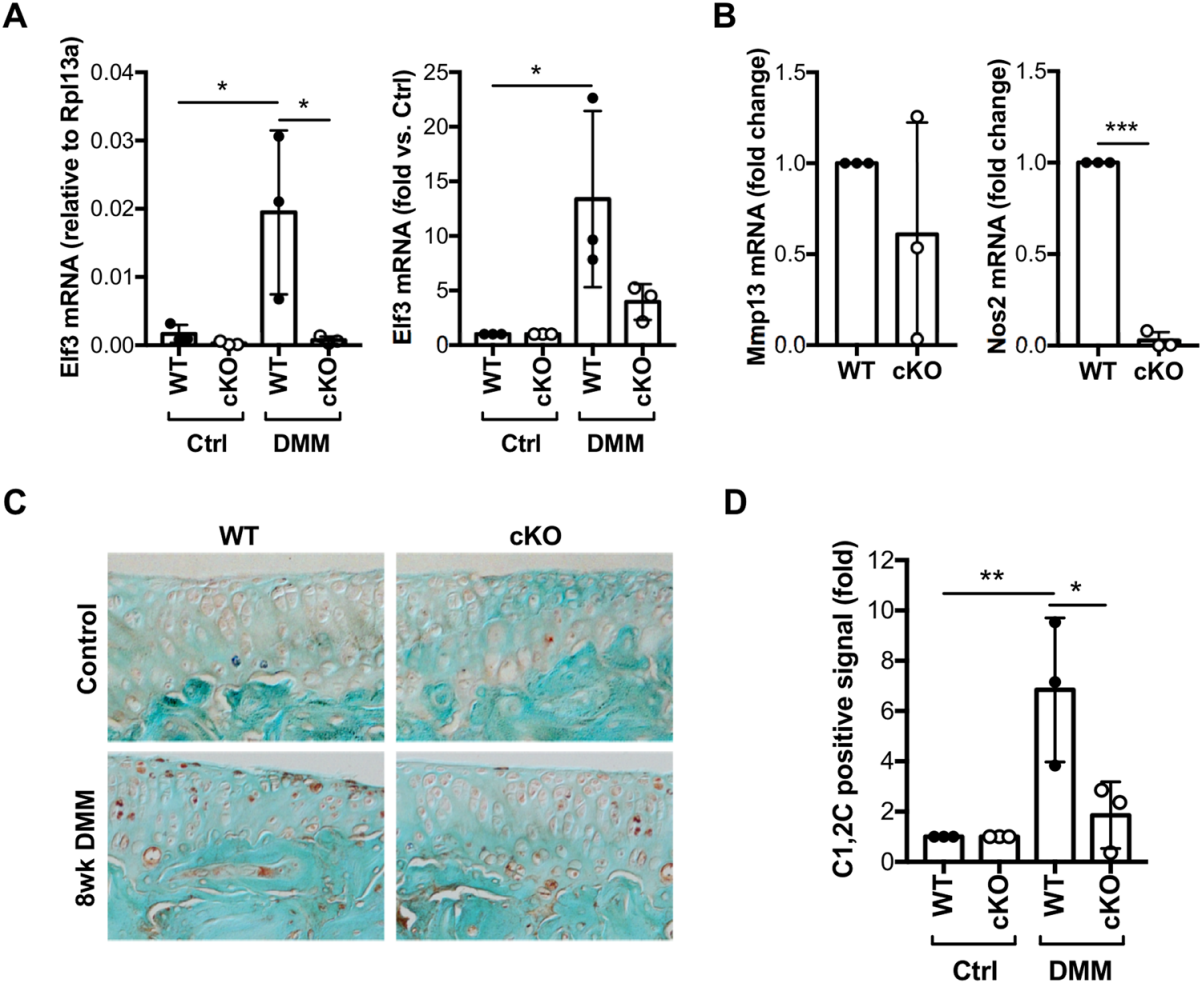
Elf3 deficiency reduces collagenase activity in mice following destabilization of the medial meniscus (DMM). RT-qPCR analyses of total RNA isolated from articular cartilage from unoperated (Ctrl) and DMM-operated (DMM) joints of WT and cKO mice (n = 3/ea) at 8 weeks after surgery showing DMM-induced *Elf3* mRNA in WT and cKO counterparts, represented as relative expression to *Rpl13a* (left) and fold-change vs. unoperated controls (right) (**A**). *Nos2* and *Mmp13* mRNA levels were assessed in cartilage isolated from DMM-operated limbs (n = 3/genotype) at 8 weeks after DMM. (**B**) Data is shown as fold change vs. WT levels (set at 1). (**C**) Representative images of unoperated (Control) or DMM-operated (8wk DMM) sections of WT and Elf3 cKO mice at 8 weeks after surgery stained with the C1, 2C antibody. (**D**) Quantification of the C1,2C-positive immunostaining at 8-weeks post-DMM revealed reduced collagenase activity in the cKO mice. Data are shown as fold-change over the immunopositive signal detected in the control knees, set at 1 (n = 3/ea). All data are shown as mean ± S.D. ***p < 0.001 by *t*-test (**B**), and *p < 0.05 and **p < 0.01 by ANOVA followed by Tukey’s post-hoc test (**A** and **D**). Comparisons with significant differences are indicated; all other comparisons were non-significant.

*Elf3* mRNA levels were found to be increased *in vivo* in DMM-operated WT cartilage samples compared to unoperated WT controls, whereas *Elf3* mRNA was virtually undetectable in both control and DMM-operated cKO cartilage samples (Fig. 4A), confirming that the protection against DMM-induced OA correlated with the loss of *Elf3* gene expression. In addition, we observed significantly decreased *Nos2* mRNA in cartilage samples obtained from DMM-operated cKO mice compared to WT counterparts (Fig. 4B). We did not detect significant changes in *Mmp13* (Fig. 4B), *Col2a1* (Supplementary Figure 4A), *Acan* (Supplementary Figure 4B) or *Timp3* (Supplementary Figure 4C) mRNA levels between WT and cKO DMM-operated samples at 8 weeks post-surgery. We next assessed collagenase activity using the C1,2 C antibody, which detects collagenase-specific cleavage epitopes on type II collagen. Immunohistochemical analyses showed reduced C1,2 C immunostaining in cKO mice compared to WT counterparts post-DMM (Fig. 4C,D). Together, our results indicate that the Elf3 cKO mice are protected against DMM-induced cartilage loss and display reduced collagenase activity *in vivo*.

### Generation and characterization of Comp-tTA;TRE-Elf3 mice

**Figure 5:**
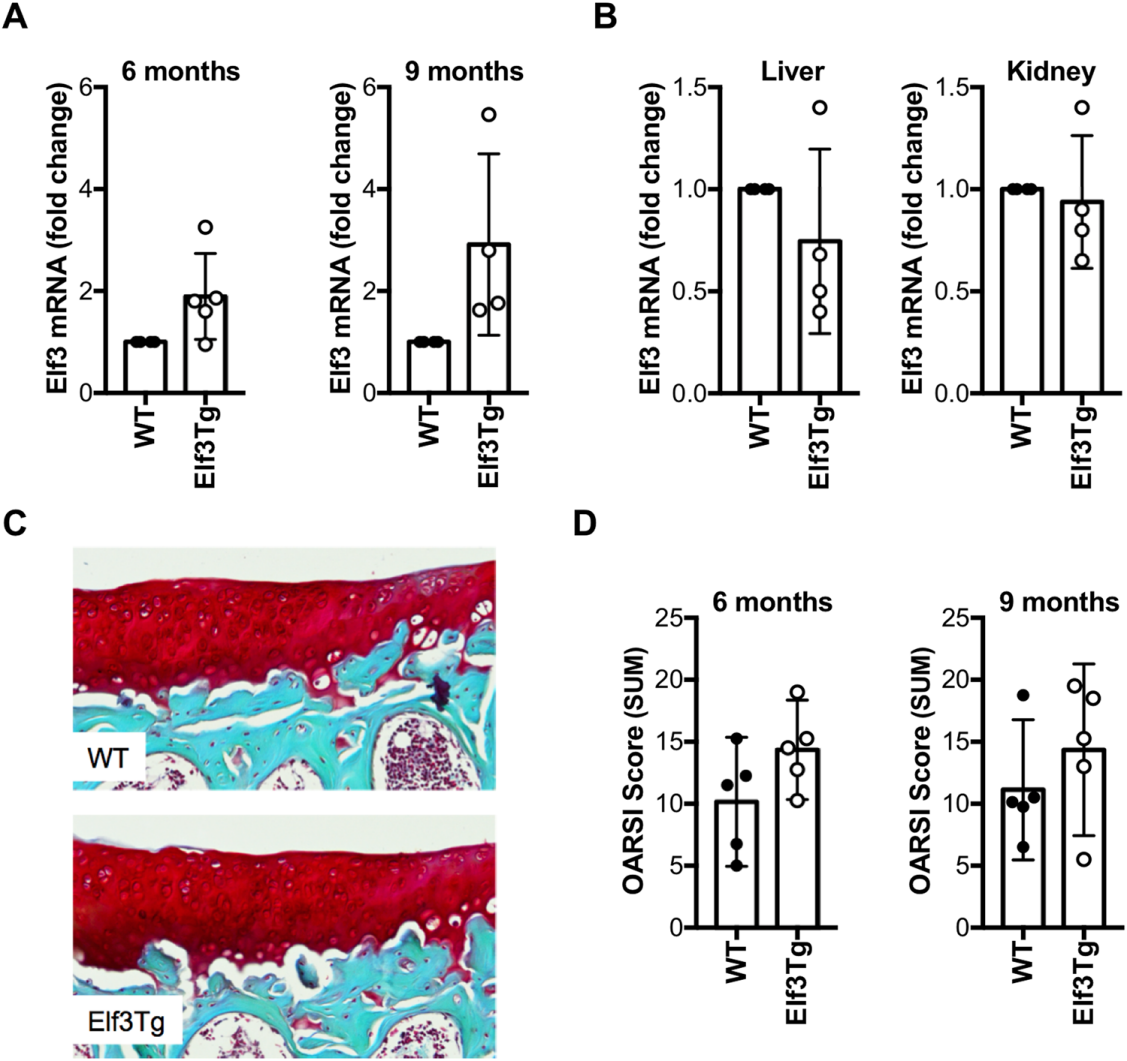
Characterization of the Elf3-Tg mice. (**A**) RT-qPCR analyses showed increased Elf3 mRNA in articular cartilage from Elf3Tg male mice after Dox withdrawal at 6 (WT = 4, Elf3Tg = 5) and 9 (n = 4/ea) months of age compared to WT littermates. Data are shown as mean ± S.D. (**B**) RTqPCR analysis of total RNA isolated from kidney and liver (n = 4/ea) showed no differences in Elf3 mRNA between WT and Elf3Tg mice at 9 months of age, after Dox withdrawal. Data are shown as mean ± S.D. (**C**) Representative images of histological sections from unoperated WT and Elf3Tg male mice at 9 months of age (8 months after Dox withdrawal) show no difference between genotypes. (**D**) OARSI cartilage degradation scoring, represented as median and C.I. (n = 5/ea), showing no significant difference between WT and Elf3Tg mice at 6 and 9 months of age (5 and 8 months after Dox withdrawal, respectively).

To determine the effects of enforced ELF3 overexpression in mouse knee joints on cartilage degradation, we generated a novel Tet-Off-inducible *Elf3*-overexpressing (*Comp*-tTA:TRE-*Elf3*, referred to as Elf3Tg) mouse strain. Pregnant females and litters were administered doxycycline until weaning, as described^21^, and post-natal *Elf3* overexpression was induced by doxycycline withdrawal in Comp-expressing tissues. No doxycycline-induced defect or gross abnormality due to the transgene was observed in any of the lines (not shown). The *Comp*tTA;TRE-*Elf3* (Elf3Tg) and TRE-Elf3 (WT) control littermates displayed comparable size and weight to *Comp*tTA and wild-type C57/B6 sex and age-matched mice at 1 and 3 months of age (not shown). We isolated total RNA from the articular cartilage of WT and Elf3Tg mice at 2, 5, and 8 months after doxycycline removal (3, 6, and 9 months of age, respectively). While we did not consistently detect changes in Elf3 mRNA in Elf3Tg mice at 2 months after doxycycline withdrawal (data not shown), increased *Elf3* mRNA was observed in the articular cartilage of Elf3Tg mice compared to WT littermates at 6 and 9 months of age (Fig. 5A). *Elf3* mRNA levels detected in kidney and liver were identical in WT and Elf3Tg mice (Fig. 5B), confirming that doxycycline withdrawal specifically upregulated Elf3 mRNA in *Comp*-expressing tissues. Finally, to assess whether increasing *Elf3* expression postnatally in joint tissues would result spontaneously in early onset of OA-like pathology, we evaluated the knee joints of 6 and 9-month-old WT and Elf3Tg mice (5 and 8 months after doxycycline removal, respectively). Safranin O staining revealed comparable structural integrity in Elf3Tg and WT littermates at 6 (not shown) and 9 months of age (Fig. 5C), which was also confirmed by the lack of significant differences in OARSI cartilage degradation scores between genotypes (Fig. 5D).

### Forced expression of *Elf3* increases cartilage degradation following destabilization of the medial meniscus

**Figure 6:**
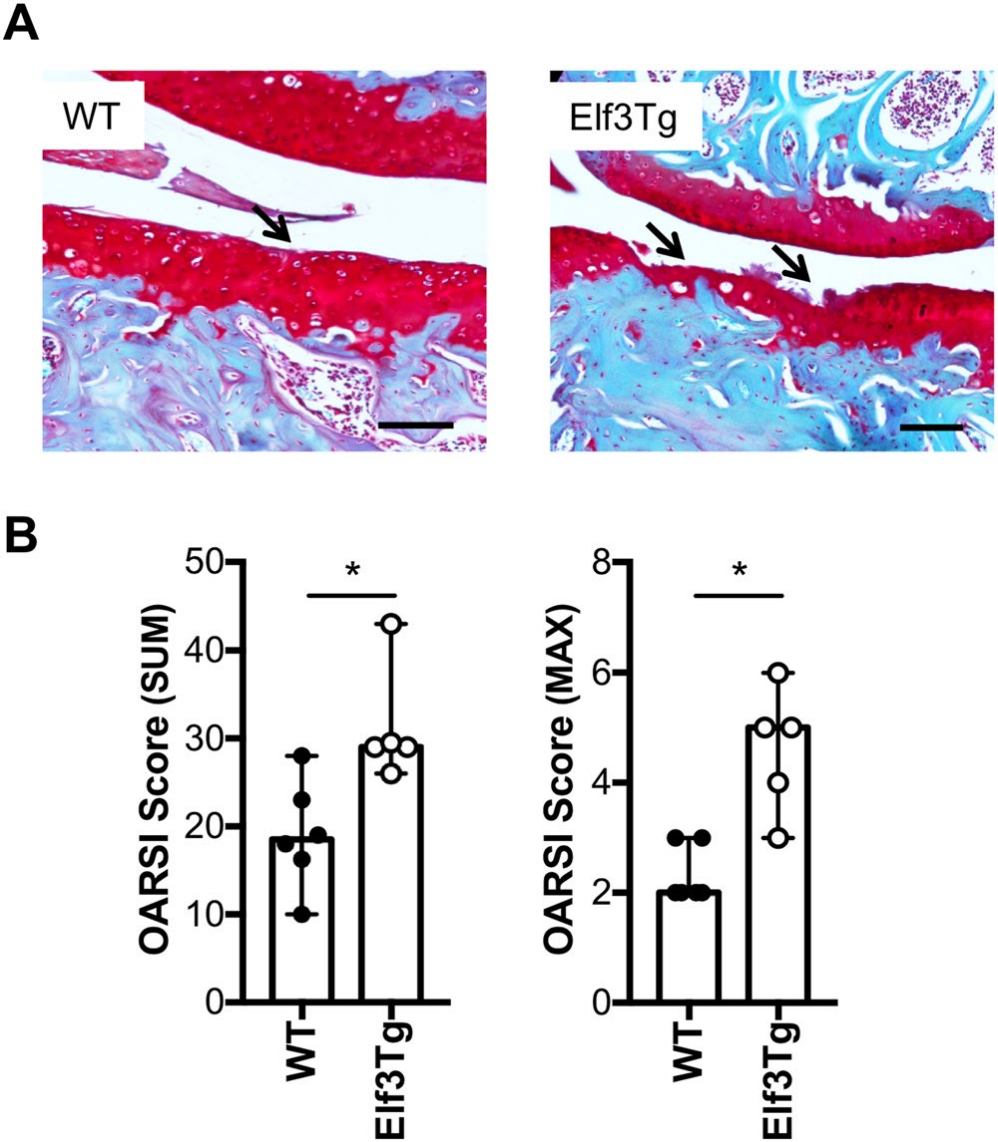
Forced expression of Elf3 increases cartilage degradation following destabilization of the medial meniscus (DMM). (**A**) Representative images of Safranin O-stained histological sections from DMM-operated 6-month-old WT and Elf3Tg male mice at 8 weeks post-DMM. The arrows indicate sites of cartilage lesions. (**B**) OARSI histological scores showing increased cartilage degradation in DMM-operated Elf3-overexpressing mice (WT = 6; Elf3Tg = 5). Data are shown as median and C.I. *p < 0.05 by Mann-Whitney test.

We next assessed whether increasing ELF3 postnatally in joint tissues would result in acceleration of post-traumatic OA by performing DMM surgeries in 6-month-old male WT and Elf3Tg mice (at 5 months after doxycycline withdrawal). Cartilage integrity was assessed in unoperated and DMM-operated WT and Elf3Tg mouse knee joints at 8 weeks post-DMM. Consistent with our previous findings (Fig. 5C,D), the Elf3Tg showed only a marginal increase in cartilage degradation scores in the unoperated limbs, further suggesting that *Elf3* overexpression is not sufficient to drive cartilage degradation. However, the DMM-operated Elf3Tg mice exhibited significantly increased cartilage degradation at 8 weeks post-DMM compared to WT counterparts (Fig. 6A,B) without inducing significant changes in osteophyte size or maturity (Supplementary Figure 5). Taken together, our results demonstrate that ELF3 contributes to cartilage degradation in OA, in part by driving MMP13 gene expression and associated collagenase activity. Our results also show that enforced post-natal expression of *Elf3* in cartilage is not sufficient to induce spontaneous OA, but increases the OA severity post-DMM, suggesting that additional biomechanical and/or inflammatory signals may be required to enable *Elf3* to transactivate genes involved in cartilage degradation.

## Discussion

OA disease involves the interplay of numerous regulators and signaling factors that in concert lead to deregulated remodeling of joint tissues. We showed previously that ELF3 mRNA and protein levels are increased in human OA cartilage, and our *in vitro* data in human and mouse primary chondrocytes provided evidence for the pivotal role of ELF3 in regulating MMP13 gene transcription^17^ and in disrupting chondrocyte anabolism^18,19^, suggesting its contribution to abnormal cartilage remodeling in OA disease. Here, we show that ELF3 contributes to increased collagenase activity and cartilage degradation *in vivo*, in a mouse model of post-traumatic OA, and that ELF3 overexpression accelerates cartilage degradation concomitant to post-traumatic OA disease.

To assess the effects of the *Elf3* deletion in OA, WT and *Elf3*-cKO littermates were subjected to the DMM model, which mimics post-traumatic OA in humans^22,23^. The DMM-operated WT mice showed enhanced cartilage damage at 8 and 12 weeks compared to the cKO mice, which had significantly lower OA scores. The lack of difference observed between the genotypes at 4 weeks post-DMM may be due to the time it requires to upregulate sufficient *Elf3* gene expression and/or activity of its target gene products, including matrix-degrading proteinases, which also require activation of pro-enzyme precursors^24^. In addition to progressive erosion of articular cartilage, OA is characterized by thickening of the subchondral bone and osteophyte formation^25^. Both *Elf3*-cKO mice and their WT littermates developed DMM-induced osteophytes, with no difference in size or maturity of osteophytes observed between genotypes, indicating that *Elf3* does not have a significant role in osteophyte formation. This result is consistent with previous reports showing that *Mmp13* knockout mice are protected from articular cartilage erosion, but not from osteophyte development^26^. The increased expression of *Elf3* mRNA in the articular cartilage of the WT DMM-operated limbs suggests that *Elf3* may be induced by mechanical stress in the absence of overt inflammation. DMM-induced expression of *Elf3* and *Mmp13* correlated with increased collagenase activity, which was reduced in cKO mice. The latter further suggests that ELF3 mediates cartilage degradation by specifically modulating MMP-13, in agreement with our previous *in vitro* results showing that other proteases are not affected by the absence of ELF3, and is consistent with the view that MMP-13 plays a central role in erosion of the collagen network, which marks OA progression^27^. However, while our observations *in vitro* support the notion that *Elf3* drives cartilage degradation via MMP-13 induction and not through other collagenase or aggrecanase-mediated events, the contribution of *Elf3* in driving the expression *in vivo* of other relevant inflammatory and catabolic genes that impact OA disease is yet to be elucidated. Similarly, with our experimental conditions we did not detect significant changes in selected anabolic genes. However, since changes in anabolism constitute a somewhat early event in OA disease and are sometimes difficult to detect reliably in cartilage with established pathology and evident structural damage, the impact of *Elf3* in the anabolic responses of OA chondrocytes *in vivo* requires further investigation.

Our data showing increased ELF3 expression in human OA cartilage and in the DMM-operated mouse cartilage, and protection of *Elf3*-cKO mice from cartilage degradation post-DMM surgery, imply that increased *Elf3* levels and/or activity may have a role in initiating or accelerating the progression of OA. To assess the effects of enforced expression of *Elf3* in adult mouse articular cartilage, we generated the *Comp*-tTA:TRE-*Elf3* (Elf3Tg) mouse strain, with inducible *Elf3* expression. We chose the Tet-Off system because of the reported advantages over other regulated gene expression systems^28,29^. While removal of doxycycline induced *Elf3* mRNA expression efficiently and specifically in the Elf3Tg cartilage, we did not observe significantly increased spontaneous or age-related cartilage degradation in the Elf3Tg mice at 6 and 9 months of age, indicating that increased *Elf3* gene expression is not sufficient to drive OA disease. Interestingly, DMM-operated Elf3Tg mice showed increased cartilage degradation at 8 weeks after surgery.

Together, these observations suggest that biomechanical challenge may be required to enhance ELF3 activity via mechanotransduction events that induce activating kinases of ELF3^17^, or to induce the expression or activity of other co-activators of ELF3, including AP-1 and NF-κB^6,30–32^. We showed previously that ELF3 synergizes with c-Fos/c-Jun to transactivate *MMP13*, and that the ELF3-induced transactivation of the *MMP13* promoter in response to IL-1β in primary chondrocytes is partially mediated by activation of MEK/ERK^17^. In addition, ELF3 has been linked to NF-κB signaling in different gene promoters and cellular contexts, being a downstream target^12,16,17,33^, a co-factor^14^, and a modulator of canonical NF-κB signaling^13^. Thus, it is conceivable that mechanical stress activates upstream signaling pathways such as MAPKs to enhance ELF3 activity, but also upregulates other transcription factors, which could then directly or indirectly interact with ELF3 to drive gene transcription in chondrocytes. Future studies should aim to further elucidate the mechanisms of action by which ELF3 accelerates OA disease progression *in vivo*, by investigating whether stress-induced MAPK activation directly phosphorylates ELF3 and increases its stability, activity, subcellular location, and DNA binding capacity *in vivo*. Furthermore, mechanical stress could increase the expression and/or activity of ELF3’s interacting partners to drive transcription in a disease-, cell-, and/or promoter-specific manner. Our findings *in vivo* suggest that ELF3 is a critical transcriptional regulator of genes required for collagen erosion during the progression of OA disease. Thus, defining the downstream targets and interacting factors of ELF3 will provide valuable insight into disease mechanisms and potentially lead to the development of novel targeted therapies for OA.

## Methods

See Supplementary Information for a detailed outline of the methods, procedures and specific materials used in this study.

### Ethics statement

All experiments were performed according to the guidelines of the American Veterinary Association and were approved by the IACUC of the Hospital for Special Surgery, and all procedures are reported following the ARRIVE guidelines^34^.

### Generation of genetically modified mice

For loss-of-function studies, we used a targeting vector obtained from the European Conditional Mouse Mutagenesis Program (EUCOMM, HTGRS6002_A_H11)^35^ to generate a novel *Elf3*^f/f^ mouse strain (deposited in Jackson labs, Elf3tm1Mote Tyrc-2J/J; stock# 030056), in which exons 2–8 of the *Elf3* gene are flanked by loxP sites. For deletion of the floxed alleles in chondrocytes, we crossed the *Elf3*^f/f^ mice with *Col2a1*-Cre mice^36,37^. Additional details about the generation and genotyping of the *Col2a1*-Cre;*Elf3*^f/f^ (*Elf3*-cKO) mice are provided in the Supplementary Methods. For gain-of-function studies, we generated the transgenic TRE-*Elf3* mice (deposited in Jackson labs, C57BL/6-Tg(tetO-Elf3)1Mote/J; stock# 030058), that express *Elf3* under the control of the tetracycline-responsive element (TRE). For this, a tet-responsive element (TRE; tetO) and a minimal CMV promoter were placed upstream of the full-length mouse *Elf3* cDNA in a transgenic vector introduced in C57/BL6 embryos. To direct expression of *Elf3* specifically in adult knee joints, we crossed the TRE-*Elf3* strain with *Comp*-tTA mice^21^. Additional details about the generation and genotyping of *Comp*tTA*;*TRE*-Elf3* (Elf3Tg) mice are provided in the Supplementary Methods.

### Destabilization of the Medial Meniscus (DMM) surgery and tissue processing post-DMM

DMM surgeries were performed, as described^20,22^, in 12-week-old male Elf3-cKO and WT littermates, or 6-month-old male Elf3Tg and WT littermates. The left knees were unoperated and served as contralateral controls. At 4, 8, and 12 weeks post-surgery, animals were euthanized and the knees collected and processed, as described^20^. Two blinded scorers assessed OA pathology^38^. The data are shown as sum and max scores. Sum scores were calculated by adding the scores of 10 sections per mouse. Osteophyte size and maturity were evaluated as described^20,26^.

### Statistical analysis

Statistical analyses were performed using GraphPad Prism 7 Software (GraphPad Software, Sand Diego, CA). Data are reported as means ± S.D. or as median and 95% C.I. (non-normally distributed data) of at least three independent experiments. Data were assessed for approximation to the Gaussian distribution using the D’Agostino-Pearson omnibus test of normality. Distributions were considered to be Gaussian if the P-value for the null hypothesis was greater than 0.05. Unpaired Student *t*-test was used to establish statistical significance between two groups. Analysis of the histological scores was performed using Mann-Whitney test. For data involving multiple groups, one-way analysis of variance (ANOVA) was performed followed by Tukey’s post-hoc test. P < 0.05 was considered significant.

### Data availability

The data that supports the findings of this study are available from the corresponding author on reasonable request.

## Electronic supplementary material

Supplementary Information
