# Supplementary material for: Elf3 Contributes to Cartilage Degradation in vivo in a Surgical Model of Post-Traumatic Osteoarthritis
Source: Sci Rep. 2018 Apr 24;8:6438. doi: 10.1038/s41598-018-24695-3 (PMC5915581; doi:10.1038/s41598-018-24695-3)

SUPPLEMENTARY INFORMATION

Elf3 Contributes to Cartilage Degradation *in vivo* in a Surgical Model of Post-Traumatic Osteoarthritis.

Elisabeth B. Wondimu[1,2], Kirsty L. Culley[1], Justin Quinn[1], Jun Chang[1], Cecilia L. Dragomir [1], Darren A. Plumb[1], Mary B. Goldring[1,2,3], Miguel Otero[1*]

From the [1]HSS Research Institute, Hospital for Special Surgery; the [2]Weill Cornell Graduate School of Medical Sciences, New York, NY 10021, USA [3]Department of Cell and Developmental Biology, Weill Cornel Medical College, New York, NY 10021, USA

[*]To whom correspondence should be addressed: Miguel Otero, Ph.D., Hospital for Special Surgery, HSS Research Institute, Room 603, 515 East 71st Street, New York, NY 10021, USA; Tel. 212-774-7561; Fax. 617-249-2373; E-mail: OteroM@hss.edu

Generation and genotyping of transgenic mouse strains:

Cartilage-specific Elf3 KO mice (Col2a1-Cre;Elf3$^{f/f}$): We generated cartilage-specific *Elf3* knockout mice with collagen type II (*Col2a1*)-driven deletion of Elf3 in the cartilage by crossing *Col2a1*-Cre mice[1] with newly generated *Elf3*$^{f/f}$ mice (Jackson labs, Elf3tm1Mote Tyrc-2J/J; stock# 030056). *Elf3*$^{f/f}$ mice were generated using a targeting construct and ES clones obtained from European Conditional Mouse Mutagenesis (EUCOMM)[2,3]. The targeted allele contains *loxP* sites upstream of *Elf3* exon 2 and downstream of *Elf3* exon 8. The vector also contained a Bact-P promoter flanked by FRT sites and inserted upstream of *Elf3*. The resulting targeting vector construct was linearized at the AsiSI site (NEB#: R0630) before electroporation into B6/Tyrc-2J ES cells and generation of chimeric mice by injection of targeted B6/Tyrc-2J ES cells into blastocysts of B6 mice (Gene Targeting Resource Center, The Rockefeller University). These mice were mated with B6/Try^{C-2J} to produce albino homozygous offspring carrying the targeted *Elf3* allele in the germline. Mice containing the floxed Elf3 and FRT sites were verified for homologous recombination within the 5' and 3' targeting arms by Southern blotting, as described[4,5]. The mice were then crossed with the FRT deleter strain from Jackson labs (B6;SJL-Tg(ACTFLPe)9205Dym/J) to delete extraneous sequences. Flipase-mediated recombination of FRT sites was verified using the following primers: *forward*: 5'-AGACCAGATCGGTGTCTTGG-3', and *reverse*: 5'-GAAATTCGGCTCCGAAAAAT-3'. The resulting *Elf3*$^{f/f}$ mice were verified by PCR using the following primers: *forward*: 5'-ACAGCAACATGACCTATGAGAAGCT-3', and *reverse*: 5'-GACAAGGGAGCTGAGCTGTCAATTT-3'. *Elf3*$^{f/f}$ mice were then crossed with *Col2a1*-Cre mice expressing Cre-recombinase under the control of the chondrocyte-specific *Col2a1* promoter[1,6]. The resulting *Col2a1*-Cre;*Elf3*$^{f/+}$ mice were crossed with *Elf3*$^{f/+}$ to produce *Col2a1*-Cre:*Elf3*$^{f/f}$ (cKO) mice. Elf3 cKO mice were identified by PCR using the following primers: *forward*: 5'-GGCGCATAACGATACCACGATA-3', and *reverse*: 5'-AGTAATACCTGCGTGGGAAGG-3'. In all experiments completed with cKO mice, Cre-negative littermates (*Elf3*$^{f/f}$, WT) were used as controls.

Tet-Off-inducible Elf3-overexpressing mice (ComptTA;TRE-Elf3): We generated mice with inducible overexpression of *Elf3* under the control of the tetracycline-responsive element (TRE) by crossing the available *Comp*-tTA mouse strain, provided by Drs. Lin Xu and Yefu Li[7], with a novel TRE-Elf3 line (Jackson labs, C57BL/6-Tg(tetO-Elf3)1Mote/J; stock# 030058). We chose to drive overexpression of *Elf3* using the *Comp* promoter because of the robust *Comp* expression in mature articular cartilage of mouse knee joints[7]. Briefly, a full sequence of *Elf3* cDNA was

cloned at the EcoRI/KpnI sites of the pTRE-tight plasmid (Clontech) containing a modified TRE[8]. The transgene construct TRE-*Elf3* was amplified by PCR, removed from the pTRE-tight plasmid vector by cutting with XhoI, separated by agarose gel electrophoresis, and purified using the Qiagen Gel Extraction Kit (Qiagen), as indicated by the manufacturer. The DNA fragment was sequenced at the Core Laboratories Center of Weill Cornell Medicine to verify the correct incorporation of *Elf3* in the pTRE-tight plasmid. The TRE-*Elf3* DNA was then incorporated into embryonic stem (ES) cells (129 strain/C57Bl/6), and injected into blastocysts of a surrogate mother mouse at The Sloan-Kettering Transgenic Mouse Facility. To identify TRE-*Elf3* mice, the TRE probe was isolated from the pTight plasmid by restriction enzyme digestion with XhoI and EcoRI, followed by separation of DNA fragments by gel electrophoresis using the Qiagen Gel Extraction Kit. Two groups of TRE-*Elf3* founder mice having high copy numbers of the transgene (based on Southern blotting assessment) were selected to breed with the *Comp*-tTA mice. In all experiments completed with *Comp*tTA;TRE-*Elf3* (Elf3Tg) mice, ComptTA negative littermates (TRE-*Elf3*, WT) were used as controls. For experiments, pregnant mothers and postnatal pups were treated with 1 mg/ml doxycycline (Sigma) in the drinking water, as described before[7]. At weaning, the doxycycline was removed to allow expression of *Elf3* mRNA. The mice were sacrificed at 3, 6, and 9 months of age to assess *Elf3* mRNA in the articular cartilage, and to perform histological evaluation of cartilage degradation. We genotyped TRE-*Elf3* mice following a well-established Southern blotting protocol, as described[4,5]. Briefly, genomic DNA was digested, amplified by PCR, separated on 2% agarose gels, and transferred overnight to nylon membranes in alkaline buffer. The DNA fragments were allowed to bind permanently to the membrane by baking at 65°C for 1 h. The membrane and filters were wetted in 2 x SSC, pre-hybridized in a solution containing 1% SDS, 6x SSC, and 10% dextran sulfate. The DNA probe containing the TRE fragment was radiolabeled with ^{32}P and denatured by heating at 100°C for 5 min. Next, the membrane was incubated with the ^{32}P-labeled TRE probe overnight. The membrane was washed in a solution containing 0.1 x SSC and 0.1% SDS, and exposed to Kodak XAR films overnight at -80°C. A representative Southern blotting membrane is shown in **Supplementary Figure 1**. The resulting double transgenic (Elf3Tg) mice were PCR-genotyped using primers that recognize the *Comp*-tTA (Forward: 5'-TGCCAACAAGGTTTTTCACTAGAGA-3', Reverse: 5'-CTCTTGATCTTCCAATACGCAACCTA-3')[7] and TRE-*Elf3* (Forward: 5'-GCTCGTTTAGTGAACCGTCAGAT-3', Reverse: 5'-ACGTTGCTGATCTCACAGGTG-3') transgenes.

Faxitron radiographic assessment:

A Faxitron x-ray machine was used to capture radiographs of limbs from cKO and WT mice at P7 and P21. The lengths of bones of the tibiae and femora were measured using ImageJ software, as described before[6,9].

Cartilage explant cultures:

Femoral heads obtained from 5- to 6-day-old WT or Elf3-cKO mice were allowed to equilibrate for 72 h in complete medium containing Dulbecco's modified Eagle's medium (DMEM)-Ham's F-12 (1/1 volume) (CellGro) and 10% fetal bovine serum (FBS), supplemented with 1% Penicillin/Streptomycin (Sigma), 1% ITS (Becton Dickinson), and 50 µg/ml L-ascorbic acid (Sigma) at 37ºC. The explants were then incubated without or with 10ng/ml of IL-1β for 6 days, with medium change every 72 h. Cartilage explants were then submerged in RNAlater (Invitrogen) overnight at 4˚C followed by RNA isolation, as described[10].

Cell culture:

Murine primary chondrocytes were isolated from 5- to 6-day-old WT and cKO pups, as described[11,12]. For experiments, passage 1 cells were treated without or with 1 ng/ml of IL-1β (R&D Systems) in serum-free medium, followed by RNA isolation.

Total RNA isolation:

Total RNA was isolated from primary mouse chondrocytes as described before[13]. We extracted total RNA from cartilage explants and from articular cartilage of control and DMM-operated mice also as described[10]. To isolate RNA from articular cartilage of mice post-DMM, articular cartilage was removed from operated or contra-lateral control mouse knees with a scalpel blade while bathed in RNAlater (Ambion) under a dissection microscope. After removing RNAlater, articular cartilage samples were homogenized in TRIzol (Invitrogen) and total RNA was extracted using a modified protocol of the miRVana mRNA isolation kit (Ambion), as described[10]. RNA integrity was assessed at the Core Laboratories Center of Weill Cornell Medicine. For RTqPCR analyses we used 100ng of total RNA with 260/280>1.8 and RIN>7.

Real-time quantitative polymerase chain reactions (RT-qPCR)

Gene amplifications were carried out using SYBR Green I-based real-time PCR, as described[12], using primers listed in **Supplementary Table 1**. The data were calculated as the ratio of each gene to *Rpl13a*, using the $2^{-\Delta\Delta Ct}$ method for relative quantification[14]. *Hprt1* and *Eef1a1* were used

as additional housekeeping genes in control experiments, but not used as normalizers in the final analyses.

Histological analysis of growth plates:

Knee joints were obtained from 1-week-old (P7), 3-week-old (P21), and 20-week-old Elf3-cKO and WT littermates and fixed, paraffin-embedded, and sectioned as described[10]. The thickness of the growth plate and heights of the proliferative and hypertrophic zones were measured using ImageJ software, on images obtained using a Nikon Ni-E microscope equipped with a DS12 bright-field camera (Nikon Instruments), as described[6]. The proliferative zone was defined as the region with flat chondrocytes stacked in longitudinal columns, and the hypertrophic zone as the region where Col10-positive chondrocytes are enlarged in size and form clusters[6].

Type X Collagen immunostaining:

Histological sections from P7 and P21 WT and cKO joints were deparaffinized in xylene, rehydrated in an ethanol series, and incubated with 2 mg/ml hyaluronidase (Sigma-Aldrich) for 30 min at 37°C to unmask antigen. After incubation with a rabbit polyclonal anti-collagen X (Col10) antibody (Abcam) overnight at 4°C, the sections were incubated with goat anti-rabbit AlexaFluor 555 conjugated secondary antibody (Cell Signaling) for 2 h at room temperature and mounted using ProLong Gold antifade medium with DAPI (Life Technologies). Control sections were incubated with a normal rabbit IgG (Santa Cruz) instead of the primary antibody (not shown). Images were captured using a Nikon Eclipse Ni-E microscope, and the mean pixel density of the hypertrophic zone was measured in at least three independent samples per genotype.

C1,2C immunohistochemistry (IHC) and analysis:

To assess differences in collagenase activity, immunostaining was performed using the Vectastain ABC rabbit IgG kit (Vector Laboratories), as described[12,15]. Briefly, paraffin-embedded sections were deparaffinized and rehydrated. After antigen retrieval (2 mg/ml hyaluronidase for 30 min at 37°C) and blocking, sections were incubated overnight with a rabbit polyclonal C1,2C antibody (IBEX). The signal was developed using NovaRED (Vector Laboratories), and sections were counterstained with Fast green. For negative controls, normal rabbit IgG (Santa Cruz) was used in place of the primary antibody. Digital images of the C1,2C-stained sections were obtained using a Nikon Eclipse Ni-E microscope (Nikon Instruments) with a DS12 bright-field camera and analyzed as described[16,17]. The total area of the region of interest (ROI) was measured in pixels.

We selected 4 to 5 ROIs per microphotograph to cover the entire cartilage surface, including articular and calcified cartilage and excluding subchondral bone. The results obtained from selected ROIs from one microphotograph were averaged to obtain one data point representative of one experiment. For quantification, the selected ROI was converted to a contrasted image using color split plugin on ImageJ. The blue channel, which showed a contrasted image with clear view of the positive and negative signal, was selected to quantify signals. The images were converted to RGB, the positive signal was selected by threshold adjustment, also as described[16,17], and the total area constituting the positive signal was quantified. The fraction denoting positive signal in a given ROI was quantified by the formula: *Positive signal = (area of positive signal)/(total area of ROI)*

Supplementary Figure 1: ***Southern blotting analysis of TRE-Elf3 transgenic mice.*** Southern blotting containing positive control DNA (50C, 10C and 1C), control wild type DNA (WT) and DNA isolated from three of the originally generated TRE-*Elf3* mice (#3, #20 and #24).

Supplementary Figure 2: ***Non-operated WT and cKO.*** **(A)** Representative images of non-operated WT and cKO mice at 4 weeks post-DMM. **(B)** OARSI histological scores of non-operated WT and cKO mice at 4, 8 and 12 weeks post-DMM showed no difference in proteoglycan content or structural integrity between genotypes.

Supplementary Figure 3: ***Osteophyte size and maturity in WT and cKO mice at 8 weeks post-DMM.*** Histological scores of DMM-operated WT and cKO mice showed no difference in osteophyte size **(A)** or maturity **(B)** between genotypes at 8 weeks post-surgery.

Supplementary Figure 4: ***RT-qPCR analyses of total RNA isolated from articular cartilage from DMM-operated joints of WT and cKO mice at 8 weeks after surgery.*** *Col2a1* **(A)**, *Acan* **(B)** and *Timp3* **(C)** mRNA levels are shown as fold change vs. WT levels (n=3/ea).

Supplementary Figure 5: ***Osteophyte size and maturity in WT and Elf3Tg mice at 8 weeks post-DMM.*** Histological scores of DMM-operated WT and Elf3Tg mice showed no difference in osteophyte size **(A)** or maturity **(B)** between genotypes at 8 weeks post-surgery.

Supplementary Table 1: ***Real-time PCR primers and Conditions.***

GENE	Primer sequences	Size (bp)	Anneal (°C)	GenBank Accesion
**Elf3**	Forward: 5'-GGCCCTCATGGCTGCCACCT-3'<br>Reverse: 5'-TTGGGATCTTGTCTGAGGTCCTGGA-3'	187	60	NM_001163131.1
**Ehf**	Forward: 5'-TCCAGTCACCTTCCAGTTGAGTC-3'<br>Reverse: 5'- CTGGGTTCTTGTCTGGGCTC-3'	148	60	NM_007914.3
**Elf5**	Forward: 5'-GGACCTAGCCACCACTTGTC-3'<br>Reverse: 5'-ATCACAGGCTGTCTGATGCTC-3'	158	60	NM_010125.3
**Mmp2**	Forward: 5'-TCGTGGCAGCCCATGAGTTCG-3'<br>Reverse: 5'-CATCGGGGGAGGGCCCATAGAG-3'	156	60	NM_008610.3
**Mmp3**	Forward: 5'-GTCCCTCTATGGAACTCCCACAGCA-3'<br>Reverse: 5'-GGACTTCTCCCCGGAGGGTGC-3'	140	60	NM_010809.2
**Mmp10**	Forward: 5'-AGCTGGCTGCTGTGCTGATCATCA-3'<br>Reverse: 5'-GAGCCACAGAACATGCAGGAGCA-3'	123	60	NM_019471.3
**Mmp12**	Forward: 5'-TTGGCCATTCCTTGGGGCTGC-3'<br>Reverse: 5'-ACTGGGGCTCCATAGAGGGACTGA-3'	139	60	NM_001320077.1
**Mmp13**	Forward: 5'-ATGGTCCAGGCGATGAAGACCCC-3'<br>Reverse: 5'-GTGCAGGCGCCAGAAGAATCTGT-3'	140	60	NM_008607.2
**Nos2**	Forward: 5'-TGCAACATGGGAGCCACAGCA-3'<br>Reverse: 5'-AGGGTGGTGCGGCTGGACTT-3'	156	60	NM_001313922.1
**Ptgs2**	Forward: 5'-CTGCTGCCCGACACCTTCAACA-3'<br>Reverse: 5'-CATTTCTTCCCCCAGCAACCCGG-3'	151	60	NM_011198.4
**Adamts4**	Forward: 5'-CCTCTGGAAGGAGGCGCCCT-3'<br>Reverse: 5'-GCGAAGCGCTTGGTTCTGCG-3'	152	60	NM_172845.3
**Adamts5**	Forward: 5'-CCTGGCGGTGGTGAAGGTGG-3'<br>Reverse: 5'-TGCCCACATAAATCCTCTCGGGTGA-3'	183	60	NM_011782.2
**Col2a1**	Forward: 5'-AATGGCACGGCTGTGTGCGA-3'<br>Reverse: 5'-AACGGGTCCCCTTGGGCCTT-3'	183	60	NM_031163.3
**Acan**	Forward: 5'-GGTCACTGTTACCGCCACTT-3'<br>Reverse: 5'-CCCCTTCGATAGTCCTGTCA-3'	175	60	NM_007424.2
**Timp3**	Forward: 5'-GAAAAGAGCGGCAGTCCCCGC-3'<br>Reverse: 5'-CCGGATCACGATGTCGGAGTTGC-3'	170	60	NM_011595.2
**Rpl13a**	Forward: 5'-AGGGGCAGGTTCTGGTATTG-3'<br>Reverse: 5'-TGTTGATGCCTTCACAGCGT-3'	120	60	NM_009438.5
**Hprt**	Forward: 5'-TCCCAGCGTCGTGATTAGCGA-3'<br>Reverse: 5'-GGGCCACAATGTGATGGCCTCC-3'	179	60	NM_013556.2
**Eef1a1**	Forward: 5'-GCCTTGGTTCAAGGGATGGA-3'<br>Reverse: 5'-ACAGTGCCAATGCCTCCAAT-3'	162	60	NM_010106.2

REFERENCES – SUPPLEMENTARY INFORMATION

1 Ovchinnikov, D. A., Deng, J. M., Ogunrinu, G. & Behringer, R. R. Col2a1-directed expression of Cre recombinase in differentiating chondrocytes in transgenic mice. *Genesis* **26**, 145-146 (2000).

2 Friedel, R. H., Seisenberger, C., Kaloff, C. & Wurst, W. EUCOMM--the European conditional mouse mutagenesis program. *Brief Funct Genomic Proteomic* **6**, 180-185, doi:10.1093/bfgp/elm022 (2007).

3 Skarnes, W. C. *et al.* A conditional knockout resource for the genome-wide study of mouse gene function. *Nature* **474**, 337-342, doi:10.1038/nature10163 (2011).

4 Weskamp, G. *et al.* Mice lacking the metalloprotease-disintegrin MDC9 (ADAM9) have no evident major abnormalities during development or adult life. *Mol Cell Biol* **22**, 1537-1544 (2002).

5 Ludwig, T. *et al.* Targeted disruption of the mouse cation-dependent mannose 6-phosphate receptor results in partial missorting of multiple lysosomal enzymes. *EMBO J* **12**, 5225-5235 (1993).

6 Hall, K. C. *et al.* ADAM17 controls endochondral ossification by regulating terminal differentiation of chondrocytes. *Mol Cell Biol* **33**, 3077-3090, doi:10.1128/MCB.00291-13 (2013).

7 Xu, L. *et al.* Intact pericellular matrix of articular cartilage is required for unactivated discoidin domain receptor 2 in the mouse model. *Am J Pathol* **179**, 1338-1346, doi:10.1016/j.ajpath.2011.05.023 (2011).

8 Gossen, M. & Bujard, H. Tight control of gene expression in mammalian cells by tetracycline-responsive promoters. *Proc Natl Acad Sci U S A* **89**, 5547-5551 (1992).

9 Bassett, J. H., van der Spek, A., Gogakos, A. & Williams, G. R. Quantitative X-ray imaging of rodent bone by Faxitron. *Methods Mol Biol* **816**, 499-506, doi:10.1007/978-1-61779-415-5_29 (2012).

10 Culley, K. L. *et al.* Mouse models of osteoarthritis: surgical model of posttraumatic osteoarthritis induced by destabilization of the medial meniscus. *Methods Mol Biol* **1226**, 143-173, doi:10.1007/978-1-4939-1619-1_12 (2015).

11 Gosset, M., Berenbaum, F., Thirion, S. & Jacques, C. Primary culture and phenotyping of murine chondrocytes. *Nat Protoc* **3**, 1253-1260, doi:10.1038/nprot.2008.95 (2008).

12 Otero, M. *et al.* E74-like factor 3 (ELF3) impacts on matrix metalloproteinase 13 (MMP13) transcriptional control in articular chondrocytes under proinflammatory stress. *J Biol Chem* **287**, 3559-3572, doi:10.1074/jbc.M111.265744 (2012).

13 Otero, M. *et al.* Human chondrocyte cultures as models of cartilage-specific gene regulation. *Methods Mol Biol* **806**, 301-336, doi:10.1007/978-1-61779-367-7_21 (2012).

14 Dussault, A. A. & Pouliot, M. Rapid and simple comparison of messenger RNA levels using real-time PCR. *Biol Proced Online* **8**, 1-10, doi:10.1251/bpo114 (2006).

15 Olivotto, E. *et al.* IKKalpha/CHUK regulates extracellular matrix remodeling independent of its kinase activity to facilitate articular chondrocyte differentiation. *PLoS One* **8**, e73024, doi:10.1371/journal.pone.0073024 (2013).

16 Girish, V. & Vijayalakshmi, A. Affordable image analysis using NIH Image/ImageJ. *Indian J Cancer* **41**, 47 (2004).

17 Varghese, F., Bukhari, A. B., Malhotra, R. & De, A. IHC Profiler: an open source plugin for the quantitative evaluation and automated scoring of immunohistochemistry images of human tissue samples. *PLoS One* **9**, e96801, doi:10.1371/journal.pone.0096801 (2014).

Supplementary Figure 1:

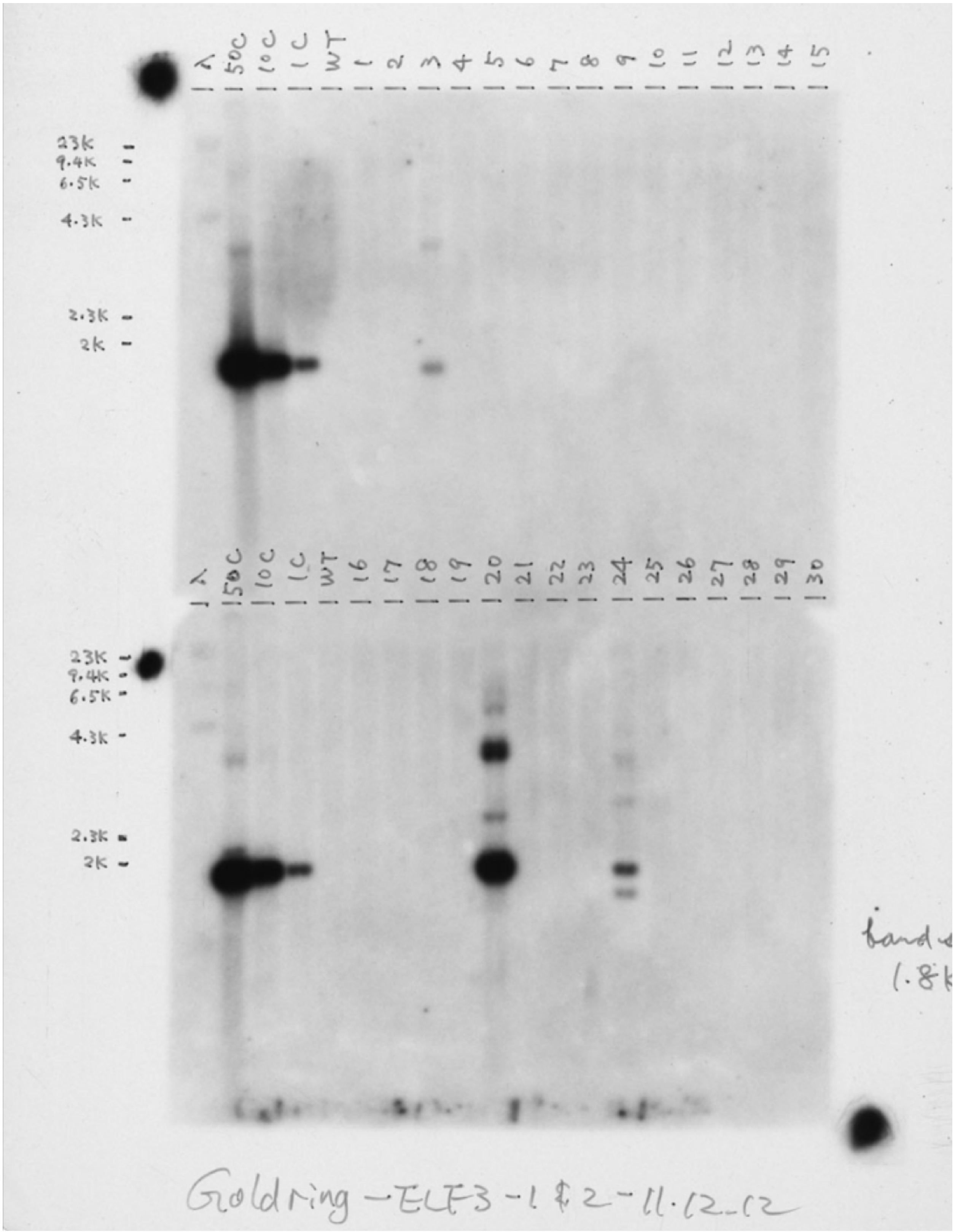

Supplementary Figure 2:

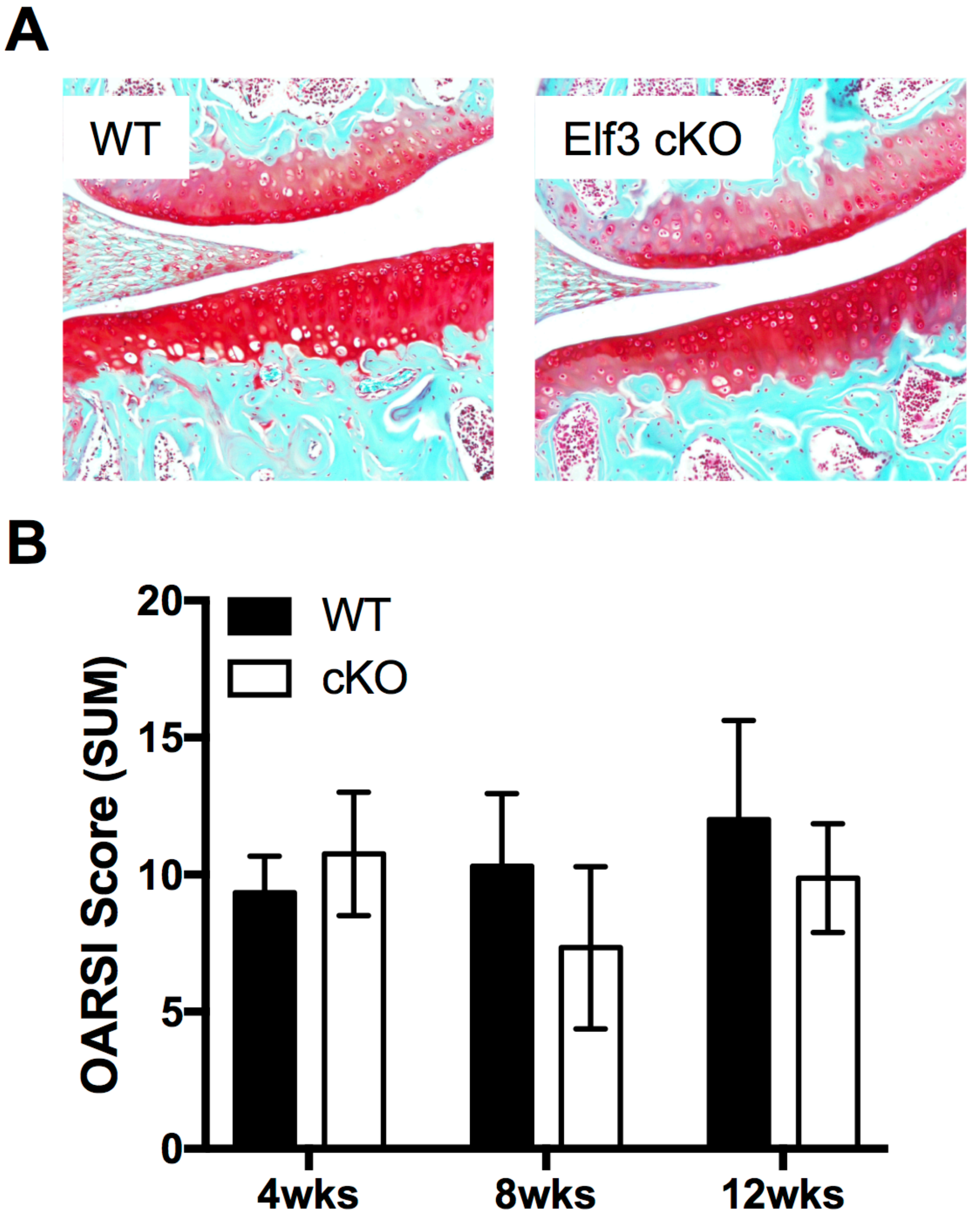

Supplementary Figure 3:

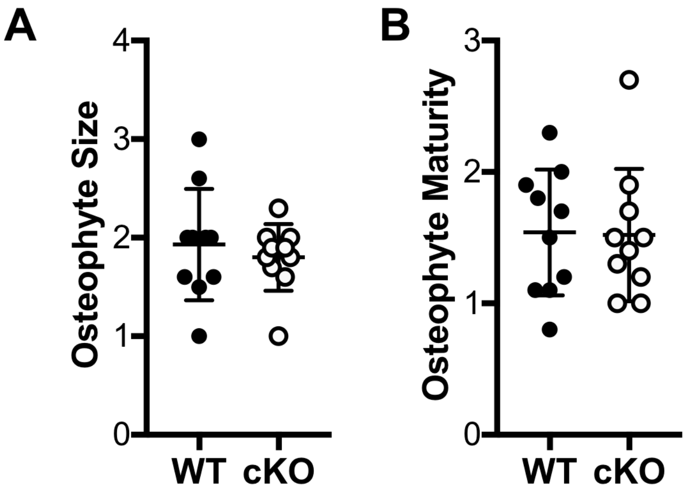

Supplementary Figure 4:

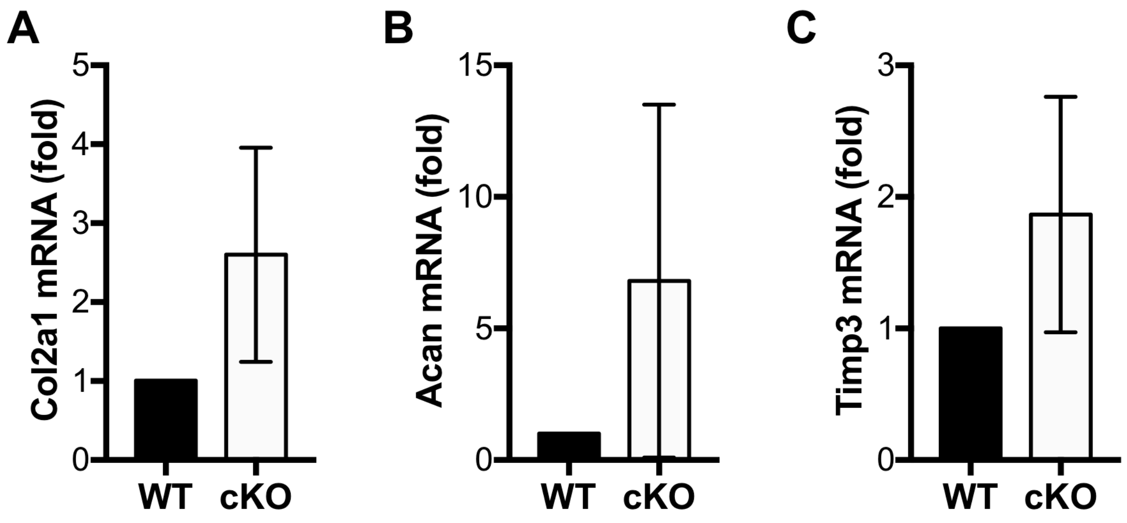

Supplementary Figure 5:

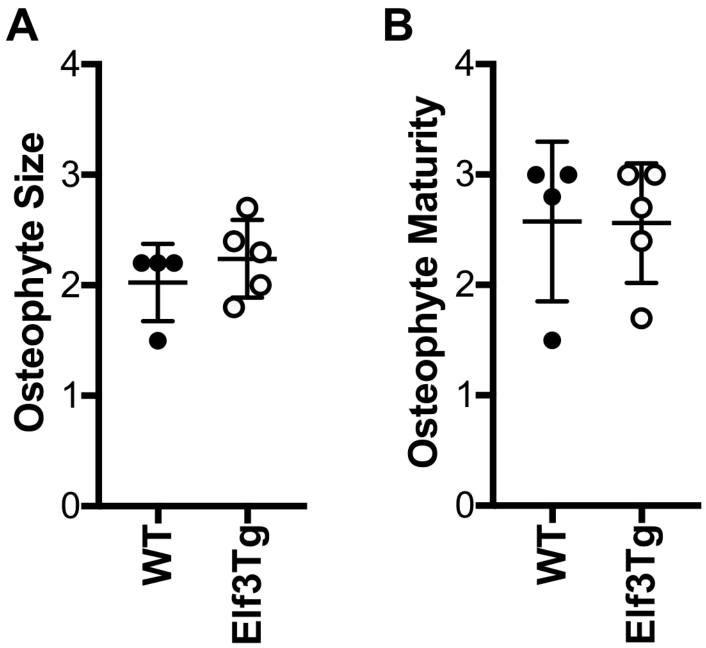

Supplement: Supplementary file 1 — Supplementary Information [file 41598_2018_24695_MOESM1_ESM.pdf]
